# Supplementary material for: Engineered Fragments of the PSMA-Specific 5D3 Antibody and Their Functional Characterization
Source: Int J Mol Sci. 2020 Sep 12;21(18):6672. doi: 10.3390/ijms21186672 (PMC7555429; doi:10.3390/ijms21186672)
Supplement: Supplementary file 1 [file ijms-21-06672-s001.pdf]

Supplementary material

Engineered fragments of the PSMA-specific 5D3 antibody and their functional characterization

Zora Novakova[1*], Nikola Belousova[1], Catherine A. Foss[2], Barbora Havlinova[1], Marketa Gresova[1], Gargi Das[1], Ala Lisok[2], Adam Prada[1], Marketa Barinkova[1], Martin Hubalek[3], Martin G. Pomper[2], and Cyril Barinka[1*]

Table S1

Type of sequence, destination vector	Amplified sequence	Forward primer (5'-3' orientation)	Reverse primer (5'-3' orientation)
antibody sequences cloned into pUC19	light VL-CL	ACCGAYATHCARATGACNCA	CTAACACTCATTCCTGTTGAAGCT
	heavy VH-CH1	GARGTNCARYTNCARCARWSNGG	CTAAATTTTCTTGTCCACCTTGG
variable domains cloned into pASK85	light VL	CCGACATCGAGCTCACCCAGAC	AGCCCGTTTTATCTCGAGCTTGG
	heavy VH	CAGCTGCAGCAGTCTGGACCTGAACTGG	GACGGTGACCGTGGTGCCTTGGCCCCAGTAGTC
Fab sequences cloned into pMT-BiP	light VL-CL	AGTAGATCTGACATCGAGCTCACCCAGACAACA	GACTAGCGCTTTACTAACACTCATTCCTGTTGAAGCTCTTGAC
	heavy VH-CH1	AGTAGATCTGAAGTTAAACTGCAGCAGTCTGGACC	GACTAGCGCTGCCGCAGTCGCGCGGTACGATTTTC
scFv construct cloned into pMT-BiP	scFv LH	ACTAGATCTGATATCCAGATGACCCAGACGACGTC	ACTGCTCGAGTTATCAGGAGCTCACTGTCAGGGTGGTG
	scFv HL	ACTAGATCTGAAGTTCAGCTGCAGCAGAGCG	ACTGCTCGAGTTATCATGCGCGCTTGATTTCAACTTTGG

Table S1. Primers used for cloning 5D3 variants.

Figure S1

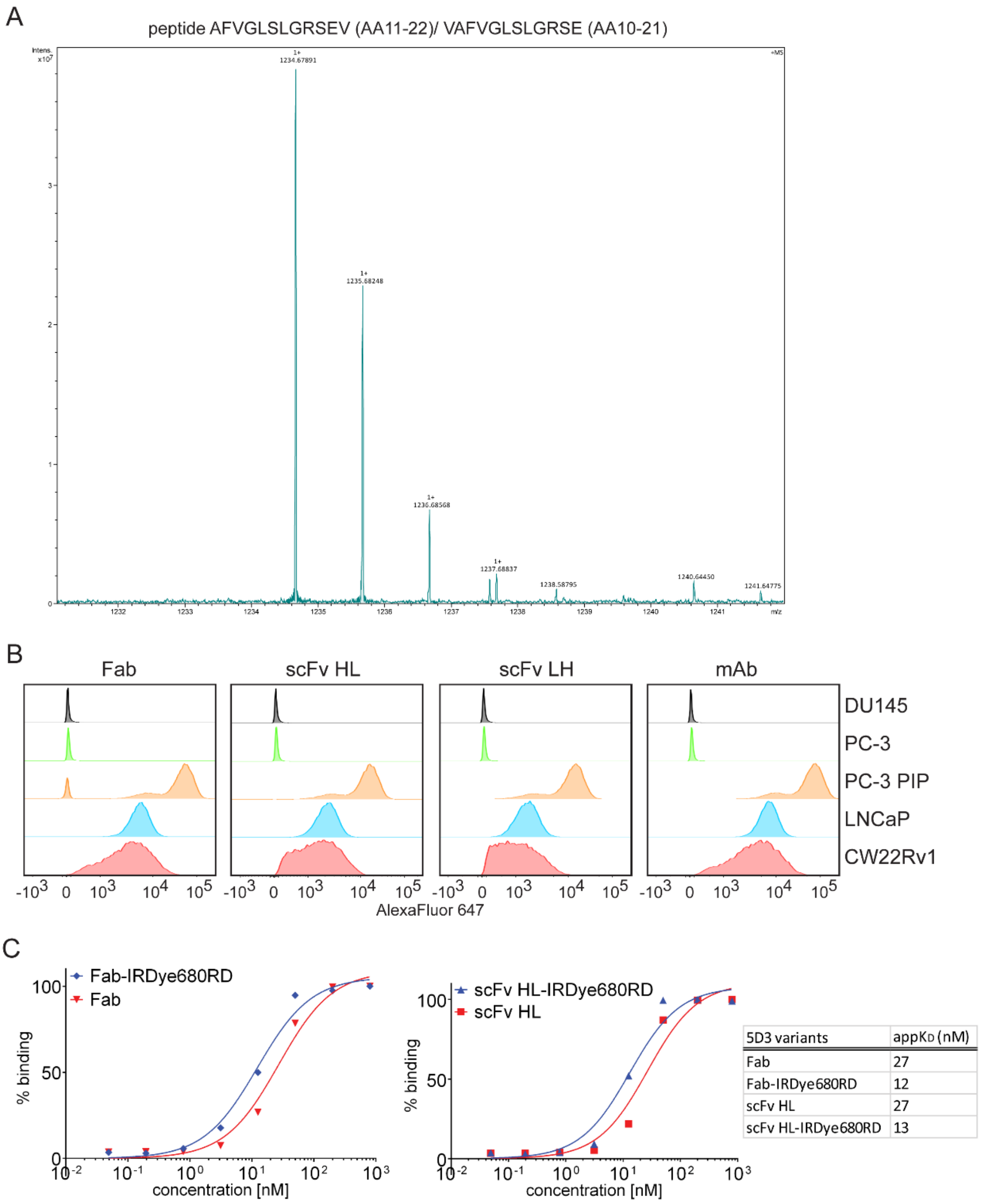

5D3 variants	appKD (nM)
Fab	27
Fab-IRDye680RD	12
scFv HL	27
scFv HL-IRDye680RD	13

Figure S1. Characterization of recombinant proteins. (**A**) Mass spectrometry data of Trypsin-digested peptides corresponding to amino acid 10 – 22 of the BiP signal sequence from the scFv HL fusion. Peptides were identified in the upper band of scFv HL separated by SDS PAGE. (**B**) Flow cytometry analysis of PSMA-positive (PC-3 PIP, LNCaP and CW22Rv1) and negative (PC-3, DU145) cells stained by 100 nM purified 5D3 variants. Each gated population represents app. 25 000 viable cells. (**C**) Affinity of conjugated antibody fragments. Apparent dissociation constants (appK_D) of recombinant proteins conjugated with IRDye680RD and their non-labeled counterparts were determined by native ELISA. Four-fold dilution series spanning the concentration range of 800 nM – 48 pM were used. Corresponding appK_D values are shown in the table.

Figure S2

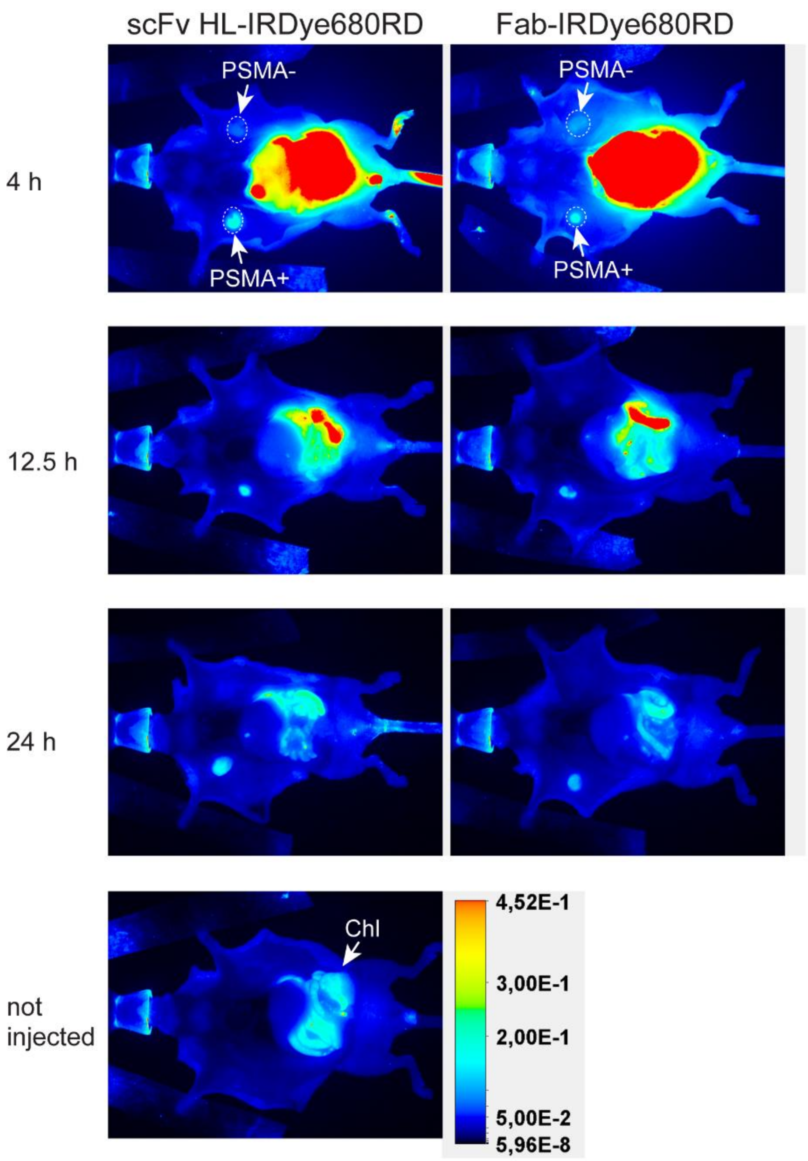

Figure S2. Distribution of Fab and scFv HL fragments in mice with xenografted PCa. PSMA-positive (PSMA+, right side) and negative (PSMA-, left side) tumors (3 – 7 mm diam., position marked by dashed circle) were formed upon subcutaneous injection of PC-3 PIP and PC-3 FLU cells, respectively. Mice were sacrificed, dissected, and scanned at various time points after tail vein injection of IRDye680RD conjugates. PSMA-positive tumors were distinguishable from negative counterparts already after 4 hours p.i. Specific signal in PSMA-positive tumors remained with the same intensity until 24 hours p.i. The fluorescent signal in the gastrointestinal tract comes from chlorophyll (Chl) present in feed. One mouse per a time point was sacrificed for each antibody fragment.

Figure S3

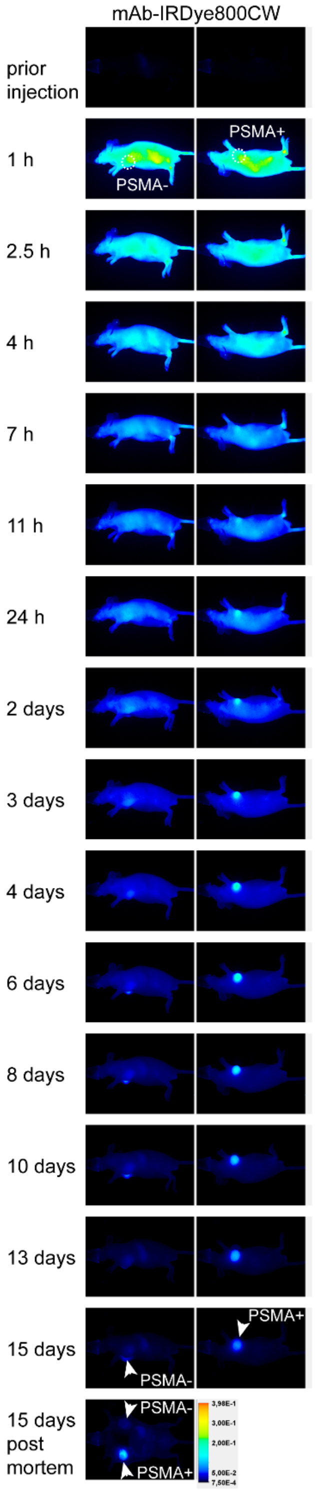

Figure S3. *In vivo* imaging of PCa xenografts by intact 5D3 mAb. Mice were inoculated by PSMA-positive (PC-3 PIP, right side) and PSMA-negative (PC-3 FLU, left side) cells. When tumors reached 4 – 5 mm in diameter (position marked by dashed circle), mice were injected intravenously with 5D3 mAb conjugated to IRDye800CW and scans taken in defined time intervals up to 15 days p.i. At the day 3 p.i., the uptake of the 5D3 conjugate was restricted to the PSMA-positive tumor only. Conjugate accumulation in the PSMA-positive tumor culminated 4 - 6 days p.i., though the signal persisted till 15 days post-injection.

Figure S4

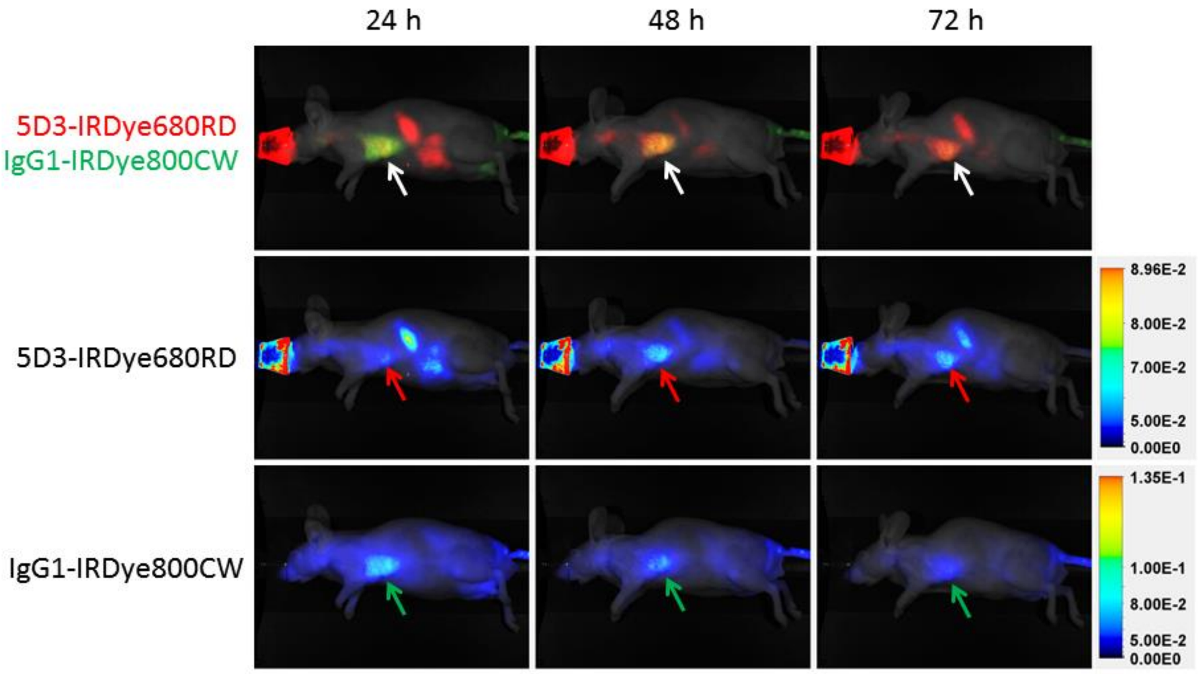

Figure S4. *In vivo* imaging of PCa including an antibody isotype control. Mice were grafted subcutaneously with PC-3 PIP cells in flank behind the left front leg. 5D3 mAb conjugated to IRDye680RD (red channel) and an isotype IgG1 control conjugated to IRDye800CW (green channel) were co-injected into the tail vein. Scans were acquired at 24 h, 48 h and 72 h p.i. with the identical exposure settings. The 5D3 conjugate was detectable in the PSMA-positive tumor already after 24 h p.i. accumulating in time up to the last time point (72 h p.i.), whereas the non-specific signal of the conjugated isotype control decreased from 24-hour time point with no intensity observed at 72 hours post-injection.

Figure S5

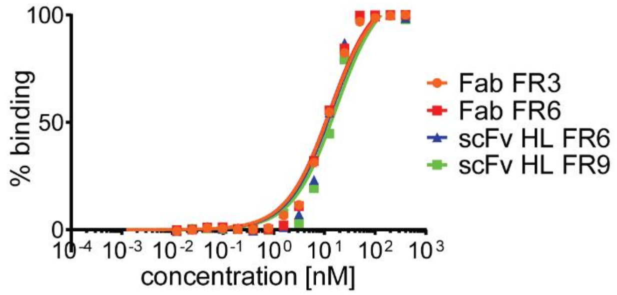

5D3 variant	upper-to-lower band ratio	appK$_D$ (nM)
Fab FR3	7:3	13
Fab FR6	1:4	13
scFv HL FR6	9:1	14
scFv HL FR9	1:4	17

Figure S5. Assessment of the effect of the uncleaved signal peptide on affinity of recombinant 5D3 fragments. Native ELISA was used to determine the affinity of Fab and scFv HL fractions containing various ratios of constructs with and without the signal peptide. Apparent K_D values revealed that affinities of all variants are virtually identical and the uncleaved signal peptide thus does not compromise fragment functionality.
